# *GmIDL2a* and *GmIDL4a*, Encoding the Inflorescence Deficient in Abscission-Like Protein, Are Involved in Soybean Cell Wall Degradation during Lateral Root Emergence

**DOI:** 10.3390/ijms19082262

**Published:** 2018-08-02

**Authors:** Chen Liu, Chunyu Zhang, Mingxia Fan, Wenjuan Ma, Meiming Chen, Fengchun Cai, Kuichen Liu, Feng Lin

**Affiliations:** College of Bioscience and Biotechnology, Shenyang Agricultural University, Shenyang 110086, China; liuchen@syau.edu.cn (C.L.); cy_zhangsyau@outlook.com (C.Z.); syndlc@outlook.com (M.F.); 20152166@stu.syau.edu.cn (W.M.); 20162164@stu.syau.edu.cn (M.C.); 2017220160@stu.syau.edu.cn (F.C.); 2017220164@stu.syau.edu.cn (K.L.)

**Keywords:** soybean, IDA-like protein, cell wall remodeling genes, lateral root development

## Abstract

The number of lateral roots (LRs) of a plant determines the efficiency of water and nutrient uptake. Soybean is a typical taproot crop which is deficient in LRs. The number of LRs is therefore an important agronomic trait in soybean breeding. It is reported that the inflorescence deficient in abscission (IDA) protein plays an important role in the emergence of *Arabidopsis* LRs. Previously, the genes which encode IDA-like (IDL) proteins have been identified in the soybean genome. However, the functions of these genes in LR development are unknown. Therefore, it is of great value to investigate the function of *IDL* genes in soybean. In the present study, the functions of two root-specific expressed *IDL* genes, *GmIDL2a* and *GmIDL4a*, are investigated. The expressions of *GmIDL2a* and *GmIDL4a*, induced by auxin, are located in the overlaying tissue, where LRs are initiated. Overexpression of *GmIDL2a* and *GmIDL4a* increases the LR densities of the primary roots, but not in the elder root. Abnormal cell layer separation has also been observed in *GmIDL2a*- and *GmIDL4a*-overexpressing roots. These results suggest that the overlaying tissues of *GmIDL2a*- and *GmIDL4a*-overexpressing roots are looser and are suitable for the emergence of the LR primordium. Further investigation shows that the expression of some of the cell wall remodeling (CWR) genes, such as xyloglucan endotransglucosylase/hydrolases, expansins, and polygalacturonases, are increased when *GmIDL2a* and *GmIDL4a* are overexpressed in hairy roots. Here, we conclude that *GmIDL2a* and *GmIDL4a* function in LR emergence through regulating soybean *CWR* gene expression.

## 1. Introduction

Root system architecture represents a key adaptability, enabling plants to cope with abiotic stresses such as drought, nutrient deficiency, and salinity, and is therefore very important for plant growth and development. The degree of root branching, determined by LR formation, influences the anchorage of plants to the soil, as well as nutrient acquisition and the efficiency of water uptake. The capacity of the root system to fulfill these functions is highly dependent on its architecture. Many monocotyledonous plants, such as maize (*Zea mays*) and rice (*Oryza sativa*), have adventitious roots, whereas dicotyledonous crops, such as cotton (*Gossypium hirsutum*) and soybean (*Glycine max*), have a primary root that repeatedly branches to generate several orders of LRs [1,2]. The degree of LR development is therefore a vital agronomic trait in crop breeding.

LRs originate from triplets of adjacent xylem pole pericycle cell pairs at regular intervals, named LR founder cells [2,3]. LR founder cells undergo several rounds of anticlinal divisions to form a single layer of primordium, consisting of small cells with equal length [4,5,6]. The subsequent rounds of divisions occur periclinally and create a two-layered lateral root primordium (LRP). Further anticlinal and periclinal divisions produce a dome-shaped LRP that divides progressively as the primary root meristem. This process is associated with auxin transport [7,8,9,10], auxin signaling [11,12], and oscillations of the auxin responses [9].

Organ emergence is another important event which restricts LR development. The LRPs are embedded within the overlaying tissue of the primary root. Therefore, to emerge, LRPs must first penetrate their parent tissues, comprising an endodermis, cortex, and epidermis. Plant cells are constrained by rigid cell walls consisting of cellulose, hemicellulose, and pectin, of which pectin is the major chemical component of the plant cell wall. The emergence of LRs and cell separation are highly coordinated. For one thing, the penetration of the LRP involves the initial cell wall loosening effected by enzymes such as xyloglucan endotransglucosylase/hydrolases (XTHs) and expansins (EXPs) [13,14]. The incompact cell walls facilitate the entrance of polygalacturonases (PGs), an important cell wall degradation enzyme which hydrolyzes pectins in cell separation events [13,14]. On the other hand, the overlaying tissue can disrupt the morphogenesis of the LRP [15].

Many biological processes are associated with cell wall loosening and degradation. Floral organ abscission happens after pollination and is controlled by the peptide IDA, which functions through its receptor leucine-rich repeat receptor-like kinases HAESA (HAE) and HAESA-LIKE2 (HSL2) [16,17]. The mutation of the *IDA* gene is associated with a deficiency in floral organ abscission in *Arabidopsis* and *Lupinus luteus* [18,19,20]. The mutant phenotype can be complemented by IDL proteins from other species [21,22]. Overexpression of *IDA* induces ectopic abscission and increases the number of rounded abscission zone (AZ) cells [23]. Microarray data shows that the IDA–HAE/HSL2 signaling module is involved in the regulation of CWR gene expression [13]. Similarly, the emergence of LRPs is dependent on cell wall dissolution. LR density is decreased in the *ida* mutant due to the constraint of overlaying the endodermal, cortical, and epidermal tissues [24]. Moreover, the IDL gene is associated with bacterial infection. *Arabidopsis* IDA6 is upregulated significantly when the plant is suffering from attacks of the bacterial *Pseudomonas syringae* pv. tomato (*Pst*) DC3000 [25]. IDL sequences are also found in the genomic sequences of *Meloidogyne* spp., which are globally deleterious pathogens of plants. The root-knot nematodes can secrete a functional IDA mimic to facilitate its infection to the host [26].

Previously, 12 *GmIDL* genes have been identified in the soybean genome, in which *GmIDL2a* and *GmIDL4a* were highly expressed in the root [27]. Moreover, the expression levels of *GmIDL2a* and *GmIDL4a* in the elder root were higher than those in root tips [27]. However, the functions of these two genes in LR development are still unknown. In the present study, we used a reversed genetics strategy to further investigate the functions of *GmIDL2a* and *GmIDL4a* in soybean LR development. We found that the expressions of *GmIDL2a* and *GmIDL4a* were induced by auxin. The overexpression of *GmIDL2a* and *GmIDL4a* can accelerate the emergence of LRPs in primary roots. The LR densities in *35S::GmIDL2a* and *35S::GmIDL4a* elder roots were not affected. Meanwhile, abnormal cell layer separation was observed in elder roots when *GmIDL2a* and *GmIDL4a* were overexpressed. Furthermore, the expressions of some of the CWR genes were increased when *GmIDL2a* and *GmIDL4a* were overexpressed. Our results enhanced the understanding of soybean LR emergence and indicated a potential use of these genes in soybean root architecture improvement.

## 2. Results

### 2.1. GmIDLs Are Highly Similar and Redundant in the Soybean Genome

**Figure 1:**
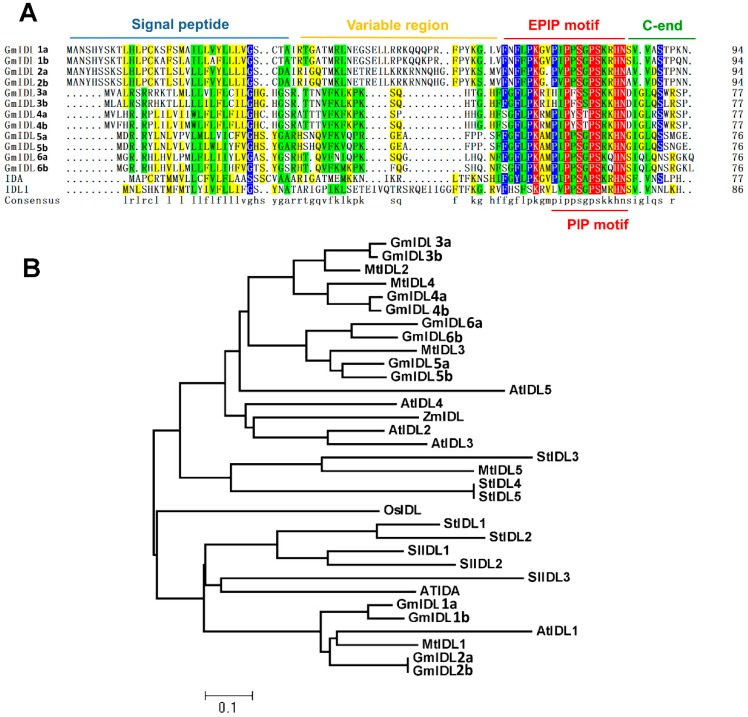
Protein sequence alignment and phylogenetic tree analysis of GmIDLs. (**A**) Alignment of soybean GmIDL proteins; and (**B**) phylogenetic tree analysis of IDL proteins in rice (OsIDL), maize (ZmIDL), tomato (SlIDL), potato (StIDL), lucerne (MtIDL), and soybean (GmIDL). IDL, IDA-like protein; EPIP, the functional motif of IDA/IDL protein.

The EPIP, the functional motif of IDA/IDL, is sufficient for IDA to play a role in floral organ abscission in *Arabidopsis* [28]. In previous reports, the soybean IDL genes were identified through BLAST searching with the use of an IDA protein sequence (NP564941) [27]. The results showed that a total of 12 proteins containing a similar motif to EPIP have been identified in the soybean genome [27]. The protein sequences are shown in Figure 1A. The twelve IDL proteins, named GmIDL1a to GmIDL6b, include putative signal peptides in their N-terminal variable regions immediately after the signal peptides and a C-end in their sequences (Figure 1A). The protein sequences of GmIDLs were very similar, indicating the redundant function of these genes.

To clarify the evolutionary relationship, we further searched for the IDL proteins in rice, maize, potato, tomato, and lucerne using the same approach. The evolutionary relationship of the twelve soybean, one rice, one maize, five lucerne, three tomato, five potato, and six *Arabidopsis* IDL proteins were displayed in the dendrogram (Figure 1B). The 12 GmIDLs were divided into two groups (Figure 1B). Group one included GmIDL1a to GmIDL2b, which shared the highest similarities with MtIDL1, IDL1, IDL, and SlIDL3, and had relatively high similarities with StIDL1, StIDL2, SlIDL1, SlIDL2, and OsIDL. Group two included GmIDL3a to GmIDL6b. In the other eight GmIDLs, GmIDL3a and GmIDL3b had the highest similarities with MtIDL2, and GmIDL4a and GmIDL4b were most similar to MtIDL4 (Figure 1B). GmIDL5a to GmIDL6b had the highest similarities with MtIDL3, AtIDL5, AtIDL4, ZmIDL, AtIDL2, and AtIDL3, respectively (Figure 1B). According to the results, we found that *GmIDL* genes were highly similar paralogous genes. These results also suggested that functional redundancy might exist in *GmIDL* genes.

### 2.2. The Expression of GmIDL2a and GmIDL4a Is Induced by Auxin and Located in the Cell Layers Embedding LRP

**Figure 2:**
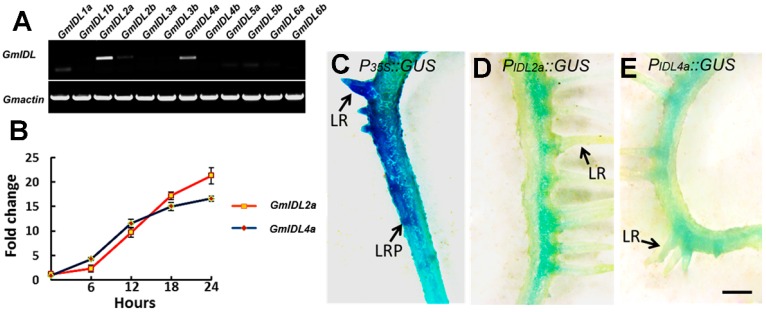
The expression of *GmIDL* genes in soybean roots. (**A**) RT-PCR analysis of *GmIDL*s in soybean roots; (**B**) relative expression of *GmIDL2a* and *GmIDL4a* under auxin treatments; (**C**–**E**) GUS histochemical staining assay of *P_35S_::GUS;* (**C**) *P_IDL2a_::GUS* and *P_IDL4a_::GUS* hairy roots under normal conditions. LR, lateral root; LRP, lateral root primordium; scale bar = 2 mm.

To identify the functional gene in soybean LR development within these 12 *GmIDLs*, total RNA was extracted from 3–4 cm soybean roots for RT-PCR assay. The results showed that *GmIDL2a*, *GmIDL2b*, and *GmIDL4a* were expressed in roots, in which *GmIDL2a* and *GmIDL4a* were highly expressed (Figure 2A). The RT-PCR results indicated potential functions of these two genes in LR development. These results were consistent with previous reporting [27]. Therefore, *GmIDL2a* and *GmIDL4a* were selected for further research.

Because GmIDL2a and GmIDL4a were proposed to be associated with LR emergence [27] and auxin has been reported to play an important role in LR development [2], we therefore tested the effect of auxin on *GmIDL* expression. The roots were treated in 1 μM IAA in MS medium for 6 to 24 h. The expression of *GmIDL2a* and *GmIDL4a* was increased after IAA treatment and acquired sharp increases after 6 h. Before 12 h, the expression of *GmIDL4a* was higher relative to *GmIDL2a*, and peaked after 18 h of treatment (Figure 2B). However, the expression of *GmIDL2a* was still increased after 18 h (Figure 2B). These results suggested that the expression of *GmIDL2a* and *GmIDL4a* was induced by auxin.

Furthermore, the expression patterns of *GmIDL2a* and *GmIDL4a* in roots were investigated using *promoter::GUS* constructs. Transgenic hairy roots harboring *P_GmIDL_::GUS* constructs were obtained from *Agrobacterium rhizogenes*-mediated transformation. Hairy roots harbouring the corresponding constructs were used for GUS histochemical staining assay. The GUS activity was detected in emerged LRs and all the cell layers of *P35S::GUS* primary roots (Figure 2C). In contrast, the promoter activities of *GmIDL2a* and *GmIDL4a* in hairy roots were only detected in one side of the cell layers where LRP emerged, and not in the other side (Figure 2D,E). The expression patterns of *GmIDL2a* and *GmIDL4a* indicated the potential functions in LR development.

### 2.3. GmIDL2a and GmIDL4a Facilitate LRP Penetration through the Overlaying Cell Layers during LR Emergence

**Figure 3:**
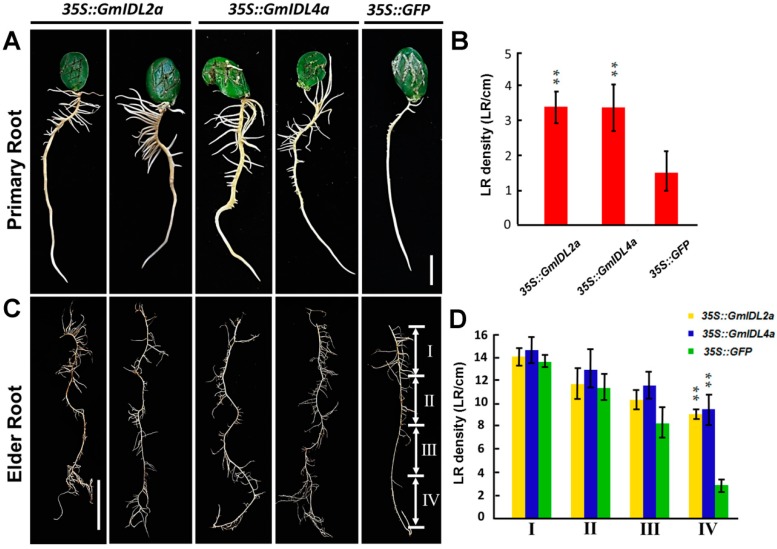
Morphological and statistical analysis of *35S::GmIDL2a*, *35S::GmIDL4a*, and *35S::GFP* primary and elder roots. (**A**) Morphologies of *35S::GmIDL2a*, *35S::GmIDL4a*, and *35S::GFP* primary hairy roots; bar = 1 cm; (**B**) LR densities of *35S::GmIDL2a*, *35S::GmIDL4a*, and *35S::GFP* primary hairy roots; (**C**) morphologies of *35S::GmIDL2a*, *35S::GmIDL4a*, and *35S::GFP* elder roots (with length more than 20 cm). I, II, III, and IV indicate four root segments; bar = 5 cm; and (**D**) LR densities of *35S::GmIDL2a*, *35S::GmIDL4a*, and *35S::GFP* in each segment of elder hairy roots. Student’s *t*-test, ** *p* < 0.01.

To investigate the functions of *GmIDL2a* and *GmIDL4a* in soybean LR development, the genes *GmIDL2a* and *GmIDL4a* were overexpressed in soybean hairy roots, driven by the *CaMV 35S* constitutive promoters. *35S::GFP* transgenic hairy roots were used as a negative control. The differences of LR densities between *35S::GFP* transgenic and untransformed roots were not significant (Table S1). After genotyping, a total of 46 *35S::GmIDL2a* and 49 *35S::GmIDL4a* transgenic hairy roots were obtained through *Agrobacterium rhizogenes*-mediated transformation, in which 34 *35S::GmIDL2a* and 31 *35S::GmIDL4a* hairy roots were, upon inspection, found to have aberrant phenotypes, respectively (Table S2, Figure 3A,B). The LRs (5–6 cm in length) were more luxuriant in *35S::GmIDL2a*- and *35S::GmIDL4a*-overexpressing roots than in the *35S::GFP* control (Figure 3A). Compared with the *35S::GFP* control, the LR densities of *35S::GmIDL2a* and *35S::GmIDL4a* hairy roots were increased significantly (Figure 3B), indicating the role of *GmIDL2a* and *GmIDL4a* in LR emergence.

To elucidate whether the increased LR densities in *35S::GmIDL2a* and *35S::GmIDL4a* transformants reflected the differences in the rates of LRP initiation or emergence, elder hairy roots with a length of more than 20 centimeters were investigated. As shown in Figure 3C, from the top of the root (segment I) to the root tip (segment IV), the hairy roots were divided into four isometric segments. Morphological analysis revealed that there were no obvious differences in LR densities between the *35S::GmIDL2a*, *35S::GmIDL4a*, and *35S::GFP* hairy roots from segment I to segment III (Figure 3C). By contrast, the LR densities in segment IV of the *35S::GmIDL2a* and *35S::GmIDL4a* hairy roots were significantly higher than those in the *35S::GFP* control (Figure 3D), indicating an accelerated LR emergence in the *35S::GmIDL2a* and *35S::GmIDL4a* hairy roots. The statistical data also supported the morphologies. These results suggested that *GmIDL2a* and *GmIDL4a* facilitate LRP penetration through the overlaying cell layers during LR emergence.

### 2.4. Overexpression of GmIDL2a and GmIDL4a Leads to Aberrant Cell Layer Separation

**Figure 4:**
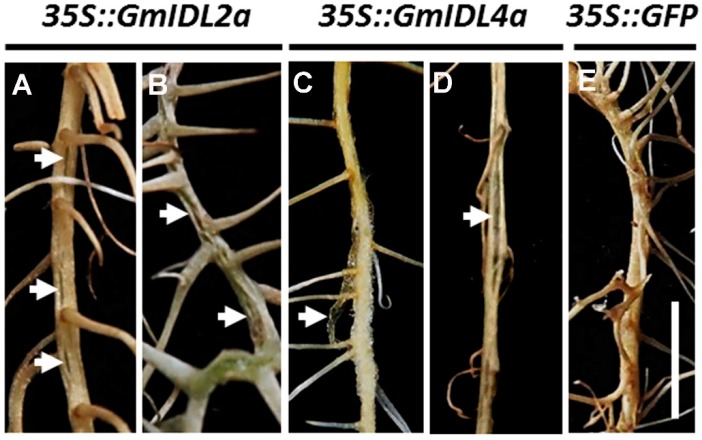
Aberrant morphologies of the *35S::GmIDL2a* and *35S::GmIDL4a* hairy roots. (**A**,**B**) Morphologies of *35S::GmIDL2a* hairy roots; (**C**,**D**) morphologies of *35S::GmIDL4a* hairy roots; and (**E**) normal root morphology of the *35S::GFP* control. Arrows indicate aberrant rifts. Scale bar = 1 cm.

As development continued, elder hairy roots with overexpression of *GmIDL2a* and *GmIDL4a* were also inspected for other changes in morphology. In *35S::GmIDL2a*- and *35S::GmIDL4a*- overexpressing roots, abnormal rifts which presumably experienced cell layer separation appeared in the elder roots. This phenotype suggested that the cell wall degradation appeared to have less control and restriction (Figure 4A–D). Infrequently, the overexpression of *GmIDL2a* and *GmIDL4a* even led to the fragmentation of the epidermis and an incompact type of the cortical cells (arrow in Figure 4C). We suspected that the adhesive force in these cell layers was insufficient to keep the epidermis adherence in place where cell wall degradation took place. As expected, these abnormal morphologies cannot be observed in the *35S::GFP* control (Figure 4E). This phenomenon reflected the functions of *GmIDL2a* and *GmIDL4a* in intercellular adhesion and overlaying cell layer separation.

**Figure 5:**
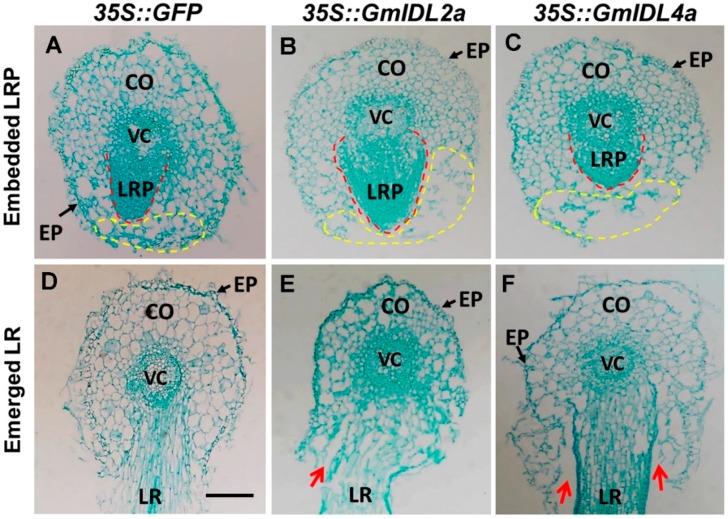
Histological sections of *35S::GFP*, *35S::GmIDL2a*, and *35S::GmIDL4a* hairy roots. (**A**–**C**) Embedded LRPs of *35S::GFP* (**A**), *35S::GmIDL2a* (**B**), and *35S::GmIDL4a* (**C**) hairy roots; (**D**–**F**) emerged LRs of *35S::GFP* (**D**), *35S::GmIDL2a* (**E**), and *35S::GmIDL4a* (**F**) hairy roots. Yellow dashed lines indicate the overlaying tissues of embedded LRPs; Red dashed lines indicate the LRPs; Red arrows indicate the abnormal cell layer separation; EP, epidermal cells; CO, cortical cells; VC, vascular cylinder; LR, lateral root. Scale bar = 200 μm.

Because LR morphogenesis was dependent on the overlaying tissues [15], we further observed the morphologies of the embedded LRPs and emerged LRs by cross section. The foreparts of the LRPs of *35S::GFP* hairy roots were narrow and sharp (Figure 5A), while the *35S::GmIDL2a* and *35S::GmIDL4a* hairy roots were wider and more obtuse (Figure 5B,C). The overlaying tissues of the embedded LRPs were compact in the *35S::GFP* control (Figure 5A). By contrast, the cell layers against the primordia were broken in the *35S::GmIDL2a* and *35S::GmIDL4a* hairy roots, suggesting an accelerated cell wall degradation (Figure 5B,C). The adhesion between the cell layers of the primary roots and emerged LRs were normal in the *35S::GFP* hairy roots (Figure 5D). However, the cell layers of the primary roots were separated from the emerged LRs when *GmIDL2a* and *GmIDL4a* were overexpressed in hairy roots (Figure 5E,F). These results indicated that the overexpression of *GmIDL2a* and *GmIDL4a* led to abnormal cell layer disintegration, which appear as rifts in their morphology (Figure 4A,B,D).

### 2.5. GmIDL2a and GmIDL4a Regulate the Expression of CWR Genes during LR Emergency

**Figure 6:**
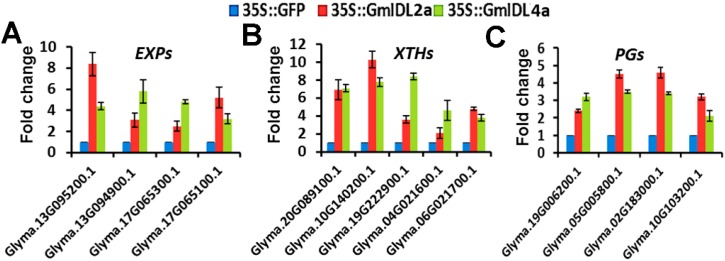
Expression of some members of *EXPs*, *XTHs*, and *PGs* genes in *35S::GmIDL2a*, *35S::GmIDL4a*, and *35S::GFP* hairy roots. (**A**) Relative expressions of *EXPs*; (**B**) relative expressions of *XTHs*; and (**C**) relative expressions of *PG*s.

Plant cell wall degradation involves initial cell wall loosening through enzymes such as EXPs and XTHs [7,8]. The loosening of the cell wall facilitates the entrance of cell wall degradation enzymes, such as PGs, which hydrolyze pectins, the major component of plant cell walls. Because the aberrant phenotypes indicated ectopic cell wall degradation, we investigated the expression levels of representative members of the CWR genes. In plants, the CWR genes *EXPs*, *XTHs*, and *PGs* belong to multigene families [29,30,31]. In the present study, four *EXPs*, five *XTHs*, and four *PGs* with high expression levels in roots were selected for further analysis. Real-time qRT-PCR results showed that the expressions of the selected *EXPs*, *XTHs*, and *PGs* in the *35S::GmIDL2a* and *35S::GmIDL4a* hairy roots were higher than those in the *35S::GFP* control (Figure 6A–C). These results suggested that *GmIDL2a* and *GmIDL4a* regulate the expression of CWR genes during LR emergence.

## 3. Discussion

### 3.1. GmIDL Genes Are Ubiquitous and Redundant in Multiple Plant Species

It has been reported that *IDA* is not only associated with floral organ abscission, but also controls the separation step of the overlaying tissues during LR emergence, suggesting the vital role of the IDA ligand in the IDA–HAE/HSL2 signaling pathway [18,32]. Besides *IDA* in *Arabidopsis*, the other five *IDL* genes have been identified through a tBLASTn search using the conserved EPIP motif of the IDA protein, which is sufficient to induce abscission through its receptor HAE/HAL2 [18,28]. Similar to *IDA*, *AtIDL1*, *AtIDL2*, and *AtIDL5* were also expressed in inflorescence; *AtIDL1* and *AtIDL4* were both expressed in roots [18]. In the present study, we performed a BLAST search in some representative monocotyledon and dicotyledon species. The results illustrated that *IDL* genes are ubiquitous in multiple plant species (Figure 1B). In soybean, there are a total of twelve *GmIDL* genes extant in the genome. The twelve GmIDL proteins can be divided into two groups. Each group contains two members which are similar in protein sequence (Figure 1A). One explanation for this phenomenon is that the diploid ancestor of soybean (*n* = 11) underwent aneuploid loss (*n* = 10) and subsequent polyploidization [33]. In the soybean genome, many genes existed as multigene families. Therefore, the functions of GmIDL proteins may be redundant in both floral organ abscission and LR emergence. Our data support this hypothesis. There are three *GmIDL* genes that were expressed in roots, of which *GmIDL2a* and *GmIDL4a* are highly expressed [Figure 2A]. Functional analysis results show that both *GmIDL2a* and *GmIDL4a* regulate the expression of CWR genes during LR emergence (Figure 4 and Figure 6).

### 3.2. IDA and IDL Act as Ligands and Are Expressed Specifically at the Site Where Cell Separation Occurs

In *Arabidopsis*, more than 1000 genes encode putative small peptides [34], suggesting that small peptides may play important roles in plant growth and development. Some small peptides function as ligands and play a role with its receptor in cell-to-cell communication through ligand–receptor interaction [24]. However, few signaling models have been identified by genetic and/or biochemical strategies [35]. The IDA–HAE/HSL2 signaling pathway is well known for its function in controlling floral organ abscission and LR emergence [18,24]. Previous studies have demonstrated that the *IDA* and *IDL* genes were expressed and restricted in both AZs and the cell layers overlaying new LRPs [18,24]. In normal conditions, plant cells attach to adjacent cells throughout their life cycle, but in some situations, it is crucial for plants to break this attachment, and thus cell separation occurs [36]. There are two consequences for cell separation. The beneficial result is the shedding of its needless organs such as flowers, fruits, or leaves; the adverse is the exposure of the separated cells to the outer environment, which puts plants at risk of pathogenic microorganism infection. Interestingly, plants have evolved some protection mechanisms, one of which is the expression pattern of the genes involved in abscission, such as *IDA* and *IDL* [18,24,25]. The expression of IDA and IDL is restricted in AZs where organ shedding occurs; consequently, this protects other tissues from pathogen infection. For LR emergence, *IDA* and *IDL* are specifically expressed in cell layers overlaying new LRPs ([18,24], Figure 2D,E). In soybean, consistent results were obtained by Tucker et al. [27]. As a result, cell wall breakdown only happens in the cells that face the LRPs. By contrast, the constitutive expression of *GmIDL2a* and *GmIDL4a* driven by *CaMV 35S* promoters in hairy roots leads to the appearance of ectopic rifts, suggesting that excessive cell wall degradation occurred (Figure 4). In the soybean genome, there are two copies of each *GmIDL* gene (Figure 1). Although they are similar in amino acid sequence, the biological functions may be different. *GmIDL2a*/*GmIDL4* and *GmIDL4a*/*GmIDL8* are the two subfamilies of the *GmIDL* gene family; however, only *GmIDL2a* and *GmIDL4a* are highly expressed in roots (Figure 2A,C).

### 3.3. GmIDL Signaling Pathways Are Involved in Cell Wall Degradation through Regulating CWR Gene Expressions

In this work, we have demonstrated that LRs of the *35S::GmIDL2a* and *35S::GmIDL4a* transgenic hairy roots find it easier to penetrate from the primary roots (Figure 3A). LRs originate from pericycle cells [37,38]; therefore, the emergence of LRs out of the overlaying tissues is the result of cell division of the LRPs [15]. In addition, the emergence of LRs is dependent on the cell layers overlaying the LRPs, because the properties of the overlaying tissues disrupt LRP morphogenesis [15]. In the present study, we found that the foreparts of the *35S::GmIDL2a* and *35S::GmIDL4a* hairy roots were wider and more obtuse. The control LRPs were narrow and sharp. Conversely, due to the restriction of the compact cell layers, LRPs in *35S::GFP* hairy roots were narrow and sharp (Figure 5A–C). For LR emergence, cell wall loosening and degradation are two critical steps [25]. In these processes, enzymes such as XTHs and EXPs are responsible for cell wall loosening, which facilitates the access of cell wall degradation enzymes such as PGs [13,14]. The PGs, important components in cell separation events, are the enzymes that hydrolyze pectins. Previous results suggest that the IDA–HAE/HAL2 signaling model is involved in the regulation of CWR genes [13]. Both *ida* mutants and *hae* mutants show deficiencies in cell wall degradation, because the expressions of CWR genes are more decreased than those of the wild type [24]. Similarly, *GmIDL2a* and *GmIDL4a* are also involved in the regulation of soybean CWR genes, which is the last step for cell wall degradation (Figure 6). However, the regulation mechanism of the IDA signaling model is not fully understood. Therefore, other factors related to signal transduction must exist in or out of this model. At present, it has been demonstrated that mitogen-activated protein kinase 4 (MKK4), MKK5, MAPK3, and MAPK6 act downstream of IDA/HAE through genetic studies and in vitro kinase assays [32]. The knotted-like homeobox genes brevipedicellus (BP)/knotted-like genes from *Arabidopsis thaliana* 1 (KNAT1) are proposed to act downstream in the IDA–HAE/HAL2 abscission pathway [39]. In conclusion, organ abscission and LR emergence are complicated physiological processes that are involved in programmed changes in cellular adhesion. More research, other than on IDA and HAE, should be performed to fully understand plant organ abscission.

## 4. Materials and Methods

### 4.1. Bioinformatic Analysis

IDL proteins were identified in the proteomes of seven species using the sequence of IDA (NP564941) and are available at the website (www.phytozome.net). Proteins which share high similarities with the EPIP motif and belong to small peptides were deemed as IDL proteins. Sequence alignment was performed using DNAMAN software. A cladogram was prepared using the software MEGA 5.0. The typical genes encoding XTH (AT4G25810), EXP (AT4G01630), and PG (AT5G14650 and AT2G41850) in soybean were identified using the *Arabidopsis* XTH (AT4G25810), EXP (AT4G01630), and PG (AT5G14650 and AT2G41850) protein sequences in the soybean genome, and then the expression data were illustrated in the BLAST result (https://phytozome.jgi.doe.gov/pz/portal.html). Genes with high expression levels in roots were selected for *CWR* gene expression level detection.

### 4.2. Soybean Root Treatment, PCR, RT-PCR, and Real-Time qRT-PCR

Soybean seeds (XIAOLIDOU) were sterilized with 70% ethanol for two minutes and then washed with sterile water five times. Furthermore, the seeds were sterilized with 0.1% HgCl_2_ for 6 min, then washed five times with sterile water for 2 min. The sterile seeds were tiled on sterile filter papers to perform the water air-drying, then incubated on 1/2 MS basal medium with the hilums upward at 28 °C under a light/dark period of 16/8 h. The germinated seeds with a root length between three and four centimeters were used for phytohormone treatment or cut down with a scalpel for RNA extraction.

For phytohormone treatment, soybean seeds were transferred to a MS medium supplemented with 1 μM IAA and treated for 6, 12, 18, and 24 h, respectively. The treated roots were harvested for RNA extraction and qRT-PCR assay.

Genomic DNA was extracted from hairy roots as described previously [40]. Total RNA was extracted using the total RNA isolation kit (Promega, Madison, WI, USA) according to the manufacturer’s recommendations. A total of 3 μg total RNA was reverse transcripted into cDNA using the reverse transcription kit (Promega, Madison, WI, USA) following the manufacturer’s instructions.

PCR and RT-PCR were performed with the gene-specific primers (Table S3) according to the amplification parameters: 95 °C for 5 min, 94 °C for 1 min, 56 °C for 1 min, and 72 °C for 30 s repeated for 30 cycles, and a final extension for 10 min at 72 °C. Reactions were carried out with Taq DNA polymerase (Takara, Dalian, China) in a Biometra Thermo cycler (TG-96; Biometra, Horsham, PA, USA), and the PCR products were analyzed on 0.8% agarose gels. Soybean actin gene (TC204137) was used as a reference gene.

Real-time qRT-PCR was performed in a 20 μL reaction system containing SYBR GREEN reaction mixture (Takara, Dalian, China), 10 μM of each primer, and 50 ng cDNA. The sequences of the primers were listed in Table S2. Amplification was carried out on a Bio-Rad Real-Time System CFX96TM C1000 thermal cycler (Hercules, CA, USA) following the manufacturer’s instructions.

### 4.3. Plasmid Construction and Soybean Transformation

The promoter fragments of *GmIDLs*, which included 2 kb upstream of the ATG of *GmIDLs*, were amplified using primer pairs P_IDL3_F/P_IDL3_R and P_IDL7_F/P_IDL7_R with *Hind*III and *Bam*HI restriction endonuclease sites at its 5′ and 3′ ends, respectively. The PCR products were cloned into the pMD19T vector (Takara, Japan) for sequencing. The correct clones were introduced into pBI121 to generate *GmIDL::GUS* constructs.

*GmIDL* encoding sequences were amplified using primer pairs GmIDL2aOEF/GmIDL2aOER and GmIDL4aOEF/GmIDL4aOER, respectively. The open reading frames of *GmIDL2a* and *GmIDL4a* were inserted into pBI121 under the control of *CaMV 35S* promoters. The constructs were transferred into *Agrobacterium rhizogenes* strain R599 for soybean transformation.

Sterile soybean seeds were cultured on 1/2 MS medium and grown at 28 °C under a light/dark period of 16/8 h. The green cotyledons were used as explants for soybean hairy root transformation. The cotyledons were cut from soybean shoots and carved the adaxial surface to increase transformation efficiency. The *Agrobacterium rhizogenes* strain harboring binary vectors were cultured to OD_600_ = 0.6–0.8 for bacterium collection. The pellet was resuspended to OD_600_ = 0.1 with MS salts supplemented with 0.1 mg/L 1-naphthlcetic acid, 2 mg/L 6-benzylaminopurine, and 10 μM acetosyringone (pH 5.2). The explants were infected by *Agrobacterium rhizogenes* for ten minutes, then the excess bacterial solution was removed by sterile filter papers. The air-dried explants were cocultured on an infection medium (MS + 10 μM acetosyringone, pH 5.2) at 25 °C in dark conditions for three days. The cocultured explants were then transferred to sterile conical flask and flushed with sterile water several times until the water was clear and pellucid. Then, the explants were transferred to hairy root-inducing medium (MS + 250 mg/L cefertaxin, pH 5.8) for hairy root induction.

### 4.4. GUS Histochemical Analysis

Hairy roots were cut from cotyledon explants and used for GUS staining assay. GUS staining was performed using the chromogenic substrate 5-bromo-4-chloro-3-indolyl-β-d-glucuronic acid (X-gluc; Bio Vectra, Oxford, CT, USA). Hairy roots were incubated with GUS staining buffer (1 mM X-gluc solution in 0.5 M EDTA, 5 mM FeK_3_(CN)_6_, 5 mM K_4_Fe(CN)_6_, and 0.1% Triton X-100 in 100 mM sodium phosphate buffer (pH 7.0)) at 37 °C for 12 h. Following alcohol decolorization, the samples were photographed under a microscope (Olympus SEX16, Tokyo, Japan).

### 4.5. Cross-Section and Microscopy

Wild-type and transgenic hairy roots were fixed in F.A.A (5% acetic acid, 5% formaldehyde solution, and 63% ethanol) at 4 °C for 48 h. After dehydration in a series of ethanol concentrations, the samples were vitrified in a series of ethanol dimethylbenzene concentrations and then embedded in paraffin. The embedded blocks were sectioned with a microtome (Leica, Solms, Germany) at a thickness of 10 μm. The sections were stained with 0.5% fast green and observed with a microscope (Olympus BX51, Tokyo, Japan).

### 4.6. Statistical Analysis

For LR density assay, a total of 30 transgenic hairy roots from each construct’s transformants were collected for statistical analysis. The hairy root density was defined as the average LR number per centimeter of taproot. The significance of difference was tested by the Student’s *t*-test (** *p* < 0.01).

## Supplementary Materials

Supplementary materials can be found at http://www.mdpi.com/1422-0067/19/8/2262/s1.

## Abbreviations

LR	Lateral root
LRP	Lateral root primordium
XTHs	Xyloglucan endotransglucosylase Hydrolases
EXPs	Expansins
PGs	Polygalacturonases
AZ	Abscission zone
CWR genes	Cell wall remodeling genes
