# Supplementary material for: GmIDL2a and GmIDL4a, Encoding the Inflorescence Deficient in Abscission-Like Protein, Are Involved in Soybean Cell Wall Degradation during Lateral Root Emergence
Source: Int J Mol Sci. 2018 Aug 2;19(8):2262. doi: 10.3390/ijms19082262 (PMC6121880; doi:10.3390/ijms19082262)
Supplement: Supplementary file 1 [file ijms-19-02262-s001.docx]

**Supplementary Materials**

*GmIDL2a* and *GmIDL4a*, Encoding the Inflorescence Deficient in Abscission-Like Protein, are Involved in Soybean Cell Wall Degradation During Lateral Root Emergence

**Table S1.** Comparison of LR densities between transgenic and untransformed roots*.

|  | **Primary root** | **Elder root** | | | |
| --- | --- | --- | --- | --- | --- |
|  |  | **I** | **II** | **III** | **IV** |
| 35S::GFP | 1.64±0.48 | 13.81±1.04 | 11.52±1.57 | 8.73±1.81 | 3.47±0.84 |
| Untransformed roots | 1.23±0.31 | 12.97±1.92 | 12.19±1.04 | 6.32±1.69 | 2.17±0.11 |

**^*^**The unit is LR/cm.

**T**able S2 Statistics of Agrobacterium rhizogenes mediated transformation.

| **Line** | **No. of hairy root** | **PCR** | **RT-PCR*** | **With phenotype** |
| --- | --- | --- | --- | --- |
| *35S::GFP* | 34 | 31 | - | 0 |
| *35S::GmIDL2a* | 47 | 46 | 44 | 34 |
| *35S:: GmIDL4a* | 51 | 49 | 46 | 31 |

*The number of hairy roots whose expression level was higher than 35S::GFP control.

**T**able S3 Primer sequences.

| **Name** | **Sequence** | **Utilization** |
| --- | --- | --- |
| GmIDL1a | F: ATGGCCAATTCTCATTATTCTAAAA | RT-PCR |
|  | R: TCAATTCTTAGGTGTGGAAGCCACC | RT-PCR |
| GmIDL1b | F: ATGGCTAATTCTCATTATTCTA | RT-PCR |
|  | R: TCAATTCTGAGGAGTGGAAGCCA | RT-PCR |
| GmIDL2a | F: ATGGCAAATTACCATTCTTCTAA | RT-PCR and qPCR |
|  | R: TCAATTATTAGGTGTTGAATCC |  |
| GmIDL2b | F: ATGGCAAATTACCATTCTTCTAA | RT-PCR |
|  | R: TCAATTATTAGGTGTTGAATCCACC |  |
| GmIDL3a | F: ATGGTAGCTCTACGTAGTAGGAG | RT-PCR |
|  | R: TTAGGGTGATCTCCAACTTTGTA |  |
| GmIDL3b | F: ATGCCATTTCCACCTCATTTTCC | RT-PCR |
|  | R: TTAGGGTGATCTCAAACTTTGTAGG |  |
| GmIDL4a | F: ATGGTTTTGCATAGGAGACCTCTGA | RT-PCR and qPCR |
|  | R: TCAGGGTGATCTCCAACTTCTTAA |  |
| GmIDL4b | F: ATGGTTTTTCATAGGAGACCTCTG | RT-PCR |
|  | R: TCAGGGTGATCTCCAACTTCTT |  |
| GmIDL5a | F: ATGGATAGAAGATACCTCAACT | RT-PCR |
|  | R: CTATGGTTCCCCCATTGAGCTT |  |
| GmIDL5b | F: ATGGATAGAAGATACCTCAACTTG | RT-PCR |
|  | R: CTATGGTTCCCCATTTGAGCTTT |  |
| GmIDL6a | F: ATGGGTAGAAGACATCTTCATGTAT | RT-PCR |
|  | R: CTATGGTTGTTTCCCCCTTGAGT |  |
| GmIDL6b | F: ATGGGTAGAAGACATCTTCATG | RT-PCR |
|  | R: CTATGGCAGTTTCCCTCTTGAGTT |  |
| GmIDL2a-OE | F: CCCAAGCTTATGGCAAATTACCATTCTT | Plasmid construction |
|  | R: CGGGATCCTCAATTATTAGGTGTTGA |  |
| GmIDL4a-OE | F: CCCAAGCTTATGGTTTTGCATAGGAGACC | Plasmid construction |
|  | R: CGGGATCCTCAGGGTGATCTCCAACTTC |  |
| P_IDL2a_ | F: TGTAATTATCTTAATTAATTGCCCAACC | Plasmid construction |
|  | R: GGAAGAGAAGCCTATAGGGATGT |  |
| P_IDL4a_ | F: AATGTGTATAAGTTTGCAAAGCAGTAA | Plasmid construction |
|  | R: GTTGTTGTTGCCAATATTTTATTATCAC |  |
| GmActin | F: CAGCATGAAAATCAAGGTGGT | RT-PCR and qPCR |
|  | R: AGGGGACCTAACGGAGAAACT |  |
| Glyma.13G095200.1 | F: ATGGCTTTTTCAAGAGTTGCAT | qPCR |
|  | R: TGATGTTCCAGAGGACACAGTGCA |  |
| Glyma.13G094900.1 | F: ATGGCTTCAACTGTCTCTTACATG | qPCR |
|  | R: AGTTTTAGCGCAAGAAGATGCT |  |
| Glyma.17G065300.1 | F: ATGGCTTCTCTTCATTCCATTACAT | qPCR |
|  | R: CTGAGGATTCCAAAGAATGGAATAGG |  |
| Glyma.17G065100.1 | F: ATGGCTTCTACCTTCTCTCGAAGC | qPCR |
|  | R: CCCTGATGGGGGTGCCATCCACA |  |
| Glyma.20G089100.1 | F: ATGGAAAAACTAATTTTCAGTGG | qPCR |
|  | R: TTTGCACCCAACTTTTCTGTAAA |  |
| Glyma.10G140200.1 | F: ATGGAAAAACTAATTTTCAGTGG | qPCR |
|  | R: CCAGCCTTGTATTTGGCGATGGTC |  |
| Glyma.19G222900.1 | F: ATGGGAAAGTTCATTTTGAGTGG | qPCR |
|  | R: GTCATGTCAAAGTGTGGTCTAGGAG |  |
| Glyma.04G021600.1 | F: ATGGCTAAAGTCATGTTTGGTT | qPCR |
|  | R: CCCTCCCTTCTTCACGCATGGAA |  |
| Glyma.19G006200.1 | F: ATGAAGAATATGAATATGAAAC | qPCR |
|  | R: TGGAAGTCTTCCATTGCTTTCCAGC |  |
| Glyma.05G005800.1 | F: GCATTGCATTGATCATTGGCATTG | qPCR |
|  | R: CCGTGCAACCAGTTTGTATAGAGA |  |
| Glyma.02183000.1 | F: ATGGCCCTCCAAAGGCATCACCTC | qPCR |
|  | R: GTTGCACTTGTAGAAAGTCAATGC |  |
| Glyma.10G103200.1 | F: ATGACACGTACATTCGTGAACATTT | qPCR |
|  | R:TGTTCTTCGCATGGTGTCTCTT |  |
